# Synaptic Paths to Neurodegeneration: The Emerging Role of TDP-43 and FUS in Synaptic Functions

**DOI:** 10.1155/2018/8413496

**Published:** 2018-04-11

**Authors:** Shuo-Chien Ling

**Affiliations:** ^1^Department of Physiology, National University of Singapore, Singapore 117549; ^2^Neurobiology/Ageing Programme, National University of Singapore, Singapore 117549; ^3^Program in Neuroscience and Behavioural Disorders, Duke-NUS Medical School, Singapore 169857

## Abstract

TAR DNA-binding protein-43 KDa (TDP-43) and fused in sarcoma (FUS) as the defining pathological hallmarks for amyotrophic lateral sclerosis (ALS) and frontotemporal dementia (FTD), coupled with ALS-FTD-causing mutations in both genes, indicate that their dysfunctions damage the motor system and cognition. On the molecular level, TDP-43 and FUS participate in the biogenesis and metabolism of coding and noncoding RNAs as well as in the transport and translation of mRNAs as part of cytoplasmic mRNA-ribonucleoprotein (mRNP) granules. Intriguingly, many of the RNA targets of TDP-43 and FUS are involved in synaptic transmission and plasticity, indicating that synaptic dysfunction could be an early event contributing to motor and cognitive deficits in ALS and FTD. Furthermore, the ability of the low-complexity prion-like domains of TDP-43 and FUS to form liquid droplets suggests a potential mechanism for mRNP assembly and conversion. This review will discuss the role of TDP-43 and FUS in RNA metabolism, with an emphasis on the involvement of this process in synaptic function and neuroprotection. This will be followed by a discussion of the potential phase separation mechanism for forming RNP granules and pathological inclusions.

## 1. Introduction

The first descriptions of ALS and FTD were provided in the late 19th century by Jean-Martin Charcot (1874) and Arnold Pick (1892), respectively, highlighting the defining features of both diseases. In the classical form of ALS, upper and lower motor neurons degenerate, leading to muscle wasting, paralysis, and eventual death typically within 5 years from disease onset. By contrast, FTD is a progressive neuronal atrophy with loss in the frontal and temporal cortices, characterized by personality and behavioral changes, as well as a gradual impairment of language skills. It is the second most common dementia after Alzheimer's disease (AD). Although seemingly different, symptoms unique to FTD and ALS can occur in the same patient, and either ALS or FTD or both can present in the same families, suggesting genetic and clinical overlaps of the two disease entities [1, 2]. In the past decade, breakthrough discoveries identifying common genetic causes and pathological hallmarks for FTD and ALS have reshaped the view that FTD and ALS are one disease continuum. Indeed, dysregulations in common molecular players, including *TARDBP* [3, 4], *FUS* [5, 6], *UBQLN2* [7], *VCP* [8], *TBK1* [9–11], *CHMP2B* [12, 13], and expanded hexanucleotide repeats within the *C9ORF72* gene [14, 15], contribute to both diseases, indicating that these ALS-FTD-linked genes can cause dysfunctions in both the motor system and cognition.

**Figure 1:**
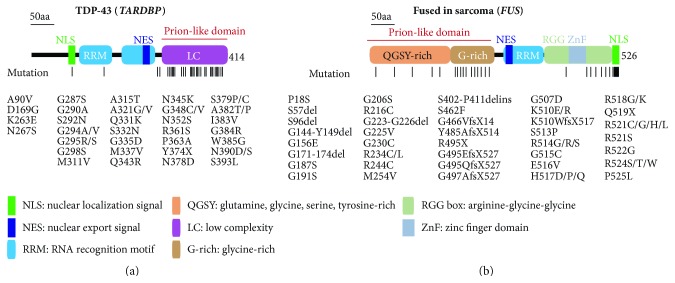
Domains and mutations for TDP-43 and FUS. Schematic and domain representation of ALS-FTD-linked mutations in TDP-43 and FUS. Lines indicate the locations of identified mutations.

Remarkably, TDP-43 (encoded by the *TARDBP* gene) and FUS (encoded by the *FUS* gene) are the major components of pathological inclusions in over 90% of all ALS and 55% of FTD cases regardless of the cause [2, 16] (Figure 1). Disease-causing mutations in genes that encode pathological hallmark proteins are commonly seen in the major adult-onset neurodegenerative diseases, underscoring the critical role of TDP-43 and FUS in driving ALS and FTD pathogenesis. Curiously, a common characteristic of TDP-43 pathology is the loss of nuclear TDP-43 with concomitant cytoplasmic TDP-43 accumulation in neurons and glia [4, 16]. This nuclear clearing supports a mechanism of disease that is at least partially driven by the loss of normal TDP-43 function in the nucleus, whereas the presence of cytoplasmic protein inclusions suggests a gain of one or more toxic properties [2, 17]. This gene-pathology-phenotype relationship implies that (1) dysfunctions in TDP-43 and FUS are able to trigger disease cascades as mutations in the *TARDBP* and *FUS* genes are causal for ALS and FTD; (2) regardless of the causes, the pathogenic process converges on TDP-43 as pathological TDP-43 inclusions are present in the majority of ALS and FTD patients (to a much lesser extent for FUS); and (3) the pathogenic mechanisms for TDP-43 and FUS are likely to be a combination of both loss-of-function and gain-of-function properties. Thus, it is critical to first understand the physiological and pathophysiological roles of TDP-43 and FUS in ALS and FTD.

**Figure 2:**
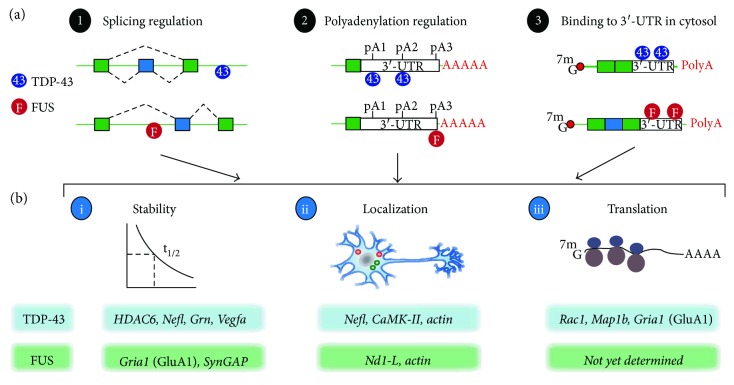
Physiological roles of TDP-43 and FUS/TLS in RNA processing. (a) 3 main functions of TDP-43 and FUS on RNA processing: splicing regulation, polyadenylation site regulation, and binding to 3′-UTR. (b) The regulation by TDP-43 and FUS influences the fates of mRNAs, stability, localization, and translation. Some of the known regulated targets are listed for TDP-43 and FUS.

Molecularly, TDP-43 and FUS are nucleic acid-binding proteins involved in the biogenesis and processing of coding and noncoding RNAs. Among the pleiotropic effects caused by TDP-43 and FUS dysfunctions, neurons that are depleted of TDP-43 and FUS, or express dominant mutations in TDP-43 and FUS, show morphological and molecular changes that indicate potential neuronal and synaptic dysfunctions. Furthermore, TDP-43 and FUS shuttle between the nucleus and the cytosol, where they may form cytoplasmic RNP granules [18] that transport within dendrites and axons. These transporting RNA granules provide a pathway to regulate synaptic strength through localized translation [19]. In fact, dendritically localized mRNAs and their localized translation is one of the underlying mechanisms that regulate the strength of synaptic transmission, a phenomenon known as synaptic plasticity, which is thought to be the molecular basis for learning and memory [20]. More recently, axonally localized mRNA in a mature neural circuit was linked to axonal survival and neurodegeneration [21]. Given the evidence that TDP-43 and FUS bind to many RNA targets important for synaptic function [22–26] (Figure 2), it is conceivable that TDP-43 and FUS can regulate synaptic plasticity through RNA transport and local translation, and dysfunction of TDP-43 or FUS may cause defects in synaptic function which will affect the health of neurons. Indeed, this “synaptic toxicity” hypothesis, in which synaptic damage is among the early events that eventually lead to neurodegeneration, has been proposed for Alzheimer's disease [27–29] and Parkinson's disease [30] and is gaining recognition in the context of FTD and ALS [31, 32]. Collectively, current evidence indicates an emerging theme, in which TDP-43- and FUS-mediated synaptic injury may lead to subsequent neurodegeneration. The general functions of TDP-43 and FUS have been extensively reviewed [2, 18, 33, 34]. Only the key functions will be highlighted in this review, with an emphasis on how TDP-43 and FUS may regulate synaptic function.

## 2. Physiological Function of TDP-43

TAR DNA-binding protein-43KDa (TDP-43) is an ubiquitously expressed 414 amino-acid protein containing two RNA recognition motifs (RRMs) followed by a glycine-rich, low-sequence-complexity prion-like domain [35, 36] (Figure 1(a)). TDP-43 was first identified as a protein binding to the transactivation response (TAR) element, a long terminal repeat of human immunodeficiency virus (HIV), resulting in the transcriptional repression of the HIV tat protein [37]. The transcriptional repressor role of TDP-43 was expanded to the promoter region of mouse *acrv1* gene, which encodes the sperm acrosomal protein SP-10, which is essential for spermatogenesis [38]. However, the exact role of TDP-43 in transcription regulation remains unresolved at this point.

In contrast, overwhelming evidence demonstrates that TDP-43 is involved in many aspects of RNA metabolism, including splicing, miRNA biogenesis, RNA transport and translation, and stress granule formation [2, 18, 33, 34]. Biochemical studies showed an interaction with a wide range of RNA-binding proteins, including numerous hnRNPs, splicing factors, and microprocessor complex (the Drosha-containing protein complex involved in microRNA processing) [26, 39, 40]. Genome-wide approaches, including a combined use of *in vivo* UV-cross-linked immunoprecipitation and high-throughput sequencing (CLIP-seq or HITS-CLIP) and other more conventional methodologies such as combining immunoprecipitation and deep sequencing (ribonucleoprotein immunoprecipitation (RIP-seq)), were used to identify the *in vivo* RNA targets for TDP-43 in mice, the human brain, and cell lines [22, 25, 26, 41, 42]. TDP-43 binds to more than 6000 RNA targets in the brain, roughly 30% of the total transcriptome. These targets can be classified into the following five categories: (1) RNA whose splicing site selection is defined by TDP-43, (2) RNA whose 3′-UTR is bound by TDP-43, (3) RNA transcripts with exceptionally long introns, (4) long noncoding RNA and small noncoding RNA, and (5) miRNA [43, 44]. Importantly, depletion of TDP-43 alters the expression and splicing of its target mRNAs, many of which are involved in synapse organization and plasticity [23, 25]. Similarly, depletion of TDP-43 also affects biogenesis of selective miRNAs [43–45] possibly via Drosha- and Dicer-dependent mechanisms [45]. Furthermore, these TDP-43-mediated miRNA changes appear to regulate neurogenesis and neurite outgrowth in neuron-like cell lines [44, 45]. Taken together, these data indicate a role of TDP-43 in regulating neuronal and synaptic functions by modulating its target coding and noncoding RNAs.

Intriguingly, a novel mechanism by which TDP-43 binds to cryptic exons to repress the splicing of these exons has been recently discovered [46, 47]. Deletions of TDP-43 resulted in the splicing of these cryptic exons to produce transcripts that are typically subjected for non-sense-mediated decay [46]. Suppression of these cryptic splicing sites by TDP-43 is critical for cell survival [46, 47]. Furthermore, these cryptic exons bound and repressed by TDP-43 are typically nonconserved and appear to differ in a cell-type-specific manner, at least in neurons and muscle cells [48]. In addition, work from the Ule group showed that TDP-43 regulates poly(A) site selection, where the distance between the TDP-43 binding site and the poly(A) site dictates the poly(A) usage in a manner similar to how splicing is regulated by TDP-43 [49]. In general, binding of TDP-43 closer to the splice site and poly(A) site represses the splicing and poly(A) usage, whereas binding of TDP-43 away from these sites enhances the splicing and poly(A) usage [41, 49]. Intriguingly, alternative usage of the poly(A) sites within the 3′-UTR of TDP-43 has been shown to autoregulate the TDP-43 expression level [50, 51]. Furthermore, as alternative polyadenylation provides cells additional means for regulating mRNA stability, localization, and translation [52], whether the TDP-43-mediated poly(A) selection shows similar tissue specificity for cryptic exon selection [48] and how TDP-43-mediated alternative polyadenylation affects neuronal and synaptic functions are exciting areas that remain to be explored.

### 2.1. TDP-43's RNA Targets in Synaptic Functions and Disease Pathogenesis

Gene expression profiling using postmortem FTD human samples with TDP-43 pathology showed downregulation of genes enriched with gene ontology (GO) terms of synapse formation, long-term potentiation, and long-term depression [53, 54]. The notion that synaptic deficit could be involved in FTD pathogenesis was further supported by the later genomic approaches obtained in various *in vivo* and *in vitro* models [22, 23, 25, 26], where many TDP-43-bound mRNAs encode neuronal and synaptic functions. The genes also involved in neurological and neurodegenerative diseases are of particular interest. These RNA transcripts include TDP-43 itself, FUS, glial excitatory amino acid transporter-2 (EAAT2, solute carrier family 1 member 2 (*SLC1A2*), or glutamate transporter 1 (GLT-1)), amyloid beta precursor protein (*APP*), presenilins, huntingtin, ataxins, synuclein, progranulin, and tau [25, 26]. Among these are ALS-linked genes whose expression levels and/or splice site selections are altered upon TDP-43 depletion [25]. These genes include FUS and EAAT2; the expression of the latter is also reduced in the FTLD-TDP brain [41]. Curiously, TDP-43 pathology is also observed in diseases caused by mutations in the above genes [55], such as Alzheimer's disease (caused by mutations in APP and presenilins) and Huntington's disease (caused by the mutations in Huntingtin), further highlighting the potential cross-talk of TDP-43 with signature proteins of other neurodegenerative diseases and the critical roles of TDP-43 in the proper functions of the nervous system.

One of the unique RNA classes bound by TDP-43 includes RNA transcripts with exceptionally long introns (average size > 100 kb), which are enriched in the brain and are often involved in synaptic activity and functions [25]. Transcripts belonging to this category that are of particular interest include neurexins 1 and 3 (*NRXN1* and *NRXN3*, resp.) and neuroligin 1 *(NLGN1)*, whose mutations are found to associate with various neurological diseases, including autism spectrum disorders [56]. Furthermore, when TDP-43 is knocked down in the central nervous system in adult mice by antisense oligonucleotides (ASOs), RNA transcripts with exceptionally long introns showed strong downregulation, suggesting a direct role of TDP-43 on maintaining the expression of these RNAs [25]. Furthermore, neurexin 3 mRNA is found to be downregulated in FTLD-TDP brains, where loss of TDP-43 nuclear staining is accompanied by cytoplasmic TDP-43 aggregation [41]. Thus, downregulation of TDP-43 strongly correlates with the reduced steady-state levels of these transcripts, providing a potential mechanism for neuronal vulnerability in TDP-43 proteinopathy. Collectively, deregulation of TDP-43 RNA targets through the loss of TDP-43 function could at least in part contribute to synaptic functions and ALS-FTD disease pathogenesis.

### 2.2. TDP-43's Role in Dendrites and Axons

#### 2.2.1. TDP-43 in Dendrites

TDP-43 has been shown to colocalize with fragile X mental retardation protein (FMRP) and Staufen, two proteins that mark transporting RNP granules and P-bodies [57], in dendrites of cultured rat hippocampal neurons [58]. Furthermore, repetitive stimulation by KCl enhanced colocalization of TDP-43, FMRP, and Staufen [58]. This early observation is reproduced by Liu-Yesucevitz et al. by further demonstrating that two ALS-linked mutations in TDP-43 (A315T and Q343R) resulted in the formation of larger RNP granules, which traveled shorter distances when neurons were stimulated with KCl [59]. This dendrite-localized TDP-43 has been shown to regulate the translation of dendritically localized mRNAs (see TDP-43 in Translational Control).

Varying TDP-43 levels in primary neurons cultured *in vitro* indicates that TDP-43 could regulate neuronal morphogenesis and spinogenesis, although the underlying mechanisms remain controversial in the literatures [60–62]. Overexpression and knockdown of TDP-43 decreases and increases spine density, respectively, in mouse primary hippocampal neurons without affecting neuronal morphology [62]. By contrast, another study showed that primary cortical neurons transfected with wild-type TDP-43 displayed increased neurite length and that expressing disease-linked mutations in TDP-43 (A315T, Q331K, and M337V) further potentiated the effect, whereas knocking down TDP-43 also increased the total neurite length [60]. However, recent work showed that both overexpression and knockdown of TDP-43 decrease dendritic branching in primary rat hippocampal and cortical neurons [61], which is consistent with the inhibition of neurite outgrowth caused by TDP-43 downregulation in neuroblastoma cell lines [63].

The majority of studies in the transgenic animals expressing disease-linked mutations in TDP-43 have been focused on the motor phenotype and ALS-like pathology [2]. Although an early report indicated a synaptic deficit in mice overexpressing human wild-type TDP-43 [64], spine density using transgenic animals expressing disease-linked mutations in TDP-43 was not examined until recently [65, 66]. While transgenic mice expressing TDP-43^A315T^ showed age-dependent reduction of dendritic spines that correlates with lower synaptic transmission within the motor cortex [66], the spine density increases in TDP-43^Q331K^ mice correlating with increased excitatory synaptic transmission [65]. Nevertheless, it is worth mentioning that a clear dose-dependent dendritic branching phenotype has been observed in *Drosophila* sensory neurons, where overexpression of fly and human TDP-43 increases and knockdown of TDP-43 decreases dendritic branching [67]. The confounding factors for these above experiments are the potential toxicity effects due to the overexpression and knockdown of TDP-43 *in vitro* [60, 68] and *in vivo* [69, 70]. Furthermore, the exact TDP-43 level cannot be precisely controlled and measured in these experimental conditions. As TDP-43 regulates a myriad of mRNAs and the TDP-43 level is exquisitely controlled, it is conceivable that different expression levels of TDP-43 may differentially affect the fates of its RNA targets. Thus, although it is clear that TDP-43 affects the structures and functions of dendritic spines, the exact mechanisms remain to be resolved.

#### 2.2.2. TDP-43 in Axons

TDP-43 RNP granules showed bidirectional microtubule-dependent transport in axons of fly motor neurons, primary rodent cortical and motor neurons, and iPSC-derived human motor neurons [71–73]. In contrast to dendritic phenotypes caused by varying the level of TDP-43 expression, the effect of TDP-43 on axonal growth and morphology is more consistent in the current literature. Overexpression of wild-type and disease-linked mutations in TDP-43 in cultured motor neurons reduces axonal length, whereas TDP-43 downregulation enhanced axonal branching [72]. The reduction of axonal growth may be due to (1) the gradual cytoplasmic accumulation of TDP-43, where the phenotype appears to correlate with ALS-linked mutations [74], and (2) destabilization of a microtubule-associated protein MAP1B/Futsch mRNA, which is bound and regulated by TDP-43 [75].

Similar to the observation in dendritic transport, RNP granules containing disease-linked mutations (G298S, A315T, and M337V) showed reduced mobility [71]. Recent work from the Holzbaur group showed that these axonal TDP-43 RNP granules behave as liquid droplets, with the granules that contain the disease-linked mutations (G298S, M337V) showing more viscous and disrupted movement [73]. Interestingly, the axonal TDP-43 RNP granules appear to contain mRNAs encoding structural components important for axonal integrity. Two known examples are neurofilament light chain *(Nefl)* [71] and microtubule-associated protein 1b *(Map1b)*, the latter of which is the homologue of *Drosophila Futsch* [75, 76]. Furthermore, a number of mRNAs encoding synaptic vesicle proteins, such as members of syntaxin and synaptotagmin, are bound by TDP-43 [25, 26, 77]. It is worth noting that the *NEFL* and synaptic vesicle proteins are among the most downregulated genes in the postmortem ALS and FTD human samples [53, 54]. Taken together, the data suggest that TDP-43 may regulate presynaptic neurons via maintaining axonal integrity as well as synaptic vesicle functions.

Two independent studies showed that *in vivo* axonal damage either by axotomy or axon ligation triggered a transient accumulation of TDP-43 at the injury sites with concomitant loss of nuclear TDP-43 [78, 79], suggesting that axonal TDP-43 localization may be a normal response for the neurons repairing themselves. Recently, Feiler et al. showed that axonal transport and presynaptic release of TDP-43 could potentially be a route of transmitting TDP-43 to the postsynaptic cells [80]. As the symptoms and TDP-43 pathology in ALS and FTD seemingly follow the anatomical connections [81, 82], it is tempting to speculate that chronic axonal damage triggers TDP-43 relocalization to axons and promotes fibril formation as demonstrated by the maturation within the RNP (ribonucleoprotein) hydrogel. As such, the prion-like spread may simply hijack the normal transportation route for propagating TDP-43 pathology [2, 83].

#### 2.2.3. TDP-43 in Translational Control

Recent work from the Shen group provides evidence that TDP-43 and FMRP coregulate dendritic translation of ~160 common targets, including Rac1, GluA1, and Map1b [84]. The results are particularly intriguing as they indicate a potential molecular link between neurodevelopmental disease and neurodegenerative disease. The notion that TDP-43 regulates protein translation is further supported by its association with RACK1 (receptor for activated C Kinase 1 protein), a known regulator for activity-dependent translation [85]. Perturbing the interaction between TDP-43 and RACK1 affects global translation, with increasing TDP-43 levels suppressing translation of multiple mRNA species [86]. Furthermore, TDP-43 represses global translation by regulating the splicing of ribosomal S6 kinase 1 (S6K1) Aly/REF-like target (SKAR), which in turn regulates the pioneering round of translation [87]. Taken together, it is apparent that TDP-43 could regulate translation via multiple mechanisms. The exact mechanisms and the contribution from each mechanism to TDP-43 regulation of axonal and dendritically localized mRNAs remain to be addressed.

## 3. Physiological Function of FUS

Sharing the structural and functional properties with TDP-43, FUS is a 526-amino acid protein containing a prion-like, low-complexity domain that is enriched with glutamine, glycine, serine, and tyrosine (Q/G/S/Y) residues [35, 88], followed by a nuclear export signal, a RNA recognition motif (RRM) domain, arginine/glycine- (R/G-) rich domains, and a zinc-finger motif and nuclear localization signal (Figure 1). FUS can bind to single- and double-stranded DNA as well as RNA and participates in a wide range of functions [2, 55, 89, 90]. FUS/TLS directly associates with RNA polymerase II (RNAP II) at the promoter region [91, 92] and is critical for the directionality of transcription [93, 94]. Furthermore, the association of FUS with U1-snRNP ensures transcription-splicing coupling [24, 95]. Independently identified as hnRNP-P2 [96], FUS is involved in alternative splicing and polyadenylation site selection [24, 93, 97, 98]. FUS shuttles between the nucleus and the cytosol [99] and is implicated in RNA localization and translation [100–102]. In addition to mRNA biogenesis and processing, FUS also associates with TATA-binding protein (TBP) and transcription factor IIIB (TFIIIB) to repress transcription by RNAP III, which transcribes small structural and catalytic RNAs [103]. FUS facilitates microRNA biogenesis by recruiting Drosha cotranscriptionally [104]. FUS is also involved in DNA damage response [105, 106] possibly via ATM (ataxia-telangiectasia-mutated)-mediated phosphorylation [107]. The link between FUS and DNA damage response is consistent with the chromosome instability and increased radiation sensitivity seen in FUS-knockout (gene-trap) cells [108–110]. Collectively, these data suggest that FUS/TLS is an important factor involved in the biogenesis and processing of coding and noncoding RNA and in maintaining genomic integrity.

### 3.1. The Role of FUS in Synaptic Functions

#### 3.1.1. FUS in Dendrites

Similar to TDP-43, FUS is involved in the transport of RNA granules in dendrites [111] using conventional kinesin and dynein as motors [101] and can move into spines in response to glutamate activation [111] through an actin-based motor protein myosin-Va [112]. Curiously, FUS appears to be able to directly associate with the postsynaptic density (PSD) based on three independent studies. In one, immunoaffinity isolation of N-methyl-D-aspartate (NMDA) receptors also pulled down FUS [113]. The association of FUS with the PSD was confirmed by isolating PSDs from mouse brains [114]. Intriguingly, the association between FUS and the PSD was shown to be increased in PSD fractions which were isolated from chemical long-term potentiation (LTP)-induced primary neurons [115], suggesting that the interaction between FUS and PSD may be regulated by neuronal activity. However, the role of FUS in the PSD is unknown.

FUS clearly is required for proper neuronal functions, as primary cortical neurons cultured from FUS-knockout mice showed altered neuronal dendritic morphology, that is, more dendritic branches, with more immature filopodia-like spines [111]. The spine morphology was rescued by overexpressing an actin-stabilization protein Nd1-L, whose mRNA is transported by FUS to the dendritic spine [100]. Reduced dendritic arborization and spine density phenotypes were observed in rodent models expressing disease-linked mutations (R521C, R521G) in FUS [116, 117], suggesting that disease-mutants may act with a dominant gain-of-function toxicity.

More recently, the Sobue group showed that depletion of FUS led to destabilization of poly(A) tails of *Gria1* mRNA, which in turn reduced the protein expression of GluA1, a subunit of the *α*-amino-3-hydroxy-5-methylisoxazole-4-propionate (AMPA) type of ionotropic glutamate receptor. AMPA receptors are typically heteromeric ion channels with a combination of 4 different subunits, GluA1–4 (encoded by *Gria1–4*) [118]. Heterodimeric GluA1/2 and GluA2/3 are the major classes of AMPA receptor pools in hippocampal neurons [119]. Among these, GluA2/3 heteromeric receptors are constitutively trafficking in and out of postsynaptic sites to maintain basal synaptic transmission, whereas the “active” GluR1/2 receptors provide a reserve pool of AMPA receptors that are stored at the “nonsynapse” sites and/or endosomal compartments. The increased trafficking and subsequent insertion of GluA1/2 into the synapses from these “nonsynapse” sites is thought to be the underlying mechanism for increasing synaptic strength during LTP process [120, 121]. Animals with FUS knockdown in the hippocampus showed hyperactivity, disinhibition, and reduced social interaction, which mimics human FTD symptoms. Remarkably, these phenotypes can be partially rescued by reintroducing *Gria1* mRNA [122]. In addition, FUS has been shown to regulate the expression SynGAP *α*2 isoform via binding and stabilizing the 3′-UTR of SynGAP mRNA. Depletion of FUS leads to reduction of SynGAP, which caused the internalization of PSD-95 into the dendritic shaft and reduced spine density. Reintroducion of SynGAP *α*2 mRNA was able to rescue the spine and behavioral abnormalities caused by FUS depletion [123]. Taken together, both the FUS expression level and disease-linked mutations were shown to affect dendritic morphology, spine density, and proper synaptic function. However, how additional mRNA targets regulated by FUS are involved in the synaptic regulation and how deregulation of these synaptic targets may relate to FTD require further investigation.

#### 3.1.2. FUS in Axons and Translational Control

Using a super-resolution microscope, Schoen et al. reveal that FUS could also localize to the presynaptic terminal [124]. Interestingly, the authors also observed that FUS is localized in the vicinity of the synaptic vesicles. The presynaptic localization of FUS is supported by the observation that ectopic FUS expression appears to travel along axons in primary cortical neurons [125]. Nevertheless, more studies are needed to resolve whether FUS is actively transported in axons. Although it is unclear how FUS may function near synaptic vesicles, it is tempting to speculate whether FUS-mediated phase separation could play a role in compartmentalization at the presynaptic terminal [126] and whether axonal FUS transport could also serve a route for prion-like spread.

In NIH/3T3 cells, FUS appears to facilitate translation for RNAs within the adenomatous polyposis coli- (APC-) containing RNP granules independent of stress-granule-like morphology [102]. Given that FUS associates with RNP granules and polysomes in the mouse brain [127], it is likely that FUS could regulate dendritic and axonal translation. Much work will be needed to decipher this possibility.

### 3.2. The Connection between TDP-43 and FUS

Biochemically, a small proportion of TDP-43 and FUS is bound in common protein complexes [40, 128]. A combination of knockdown, overexpression, and rescue studies of TDP-43 and FUS in *Drosophila* and zebrafish support the notion that TDP-43 and FUS function in common RNA maturation pathways [129–131], where FUS appears to act downstream of TDP-43 and disease-linked mutations in TDP-43 and FUS potentiate the toxicity potentially through enhanced interactions [40]. Recent work by Shiihashi et al. showed that transgenic mice expressing wild-type human TDP-43 potentiates toxicity in NLS-deleted FUS transgenic mice [132], further supporting the notion that TDP-43 and FUS may act on the same cascade. On a molecular level, genome-wide RNA analyses have identified a requirement for TDP-43 or FUS for maturation of a convergent set of pre-mRNAs with exceptionally long introns in mouse brains [24] and >25% of genes with respect to their expression in primary cortical neurons [23], supporting a common loss-of-function pathway as one component from the misregulation of TDP-43 or FUS. However, it remains elusive what the critical deregulated pathways are and how they contribute to ALS and FTD pathogenesis.

## 4. Potential Mechanisms for TDP-43 and FUS-Containing RNA Granules and Pathological Inclusions: Liquid Demixing and Phase Separation

One of the key questions is why and how TDP-43 and FUS are prone to form pathological inclusions in ALS and FTD. Recent work has shown that full-length TDP-43 forms amyloid oligomers that are toxic to neurons [133], possibly through head-to-tail interactions [134]. Furthermore, based on structural organization, TDP-43 and FUS can be separated into nucleic acid binding and the intrinsically disordered prion-like domains, the latter of which contain low-complexity amino acid compositions such as Q/N-rich in TDP-43 or Q/G/S/Y-rich in FUS (Figure 1). The low-complexity prion-like domains are shown to self-assemble and form liquid droplets. Therefore, the combination of RNA-binding and self-assembly domains may provide a molecular mechanism for how RNA granules are formed [35, 135].

**Figure 3:**
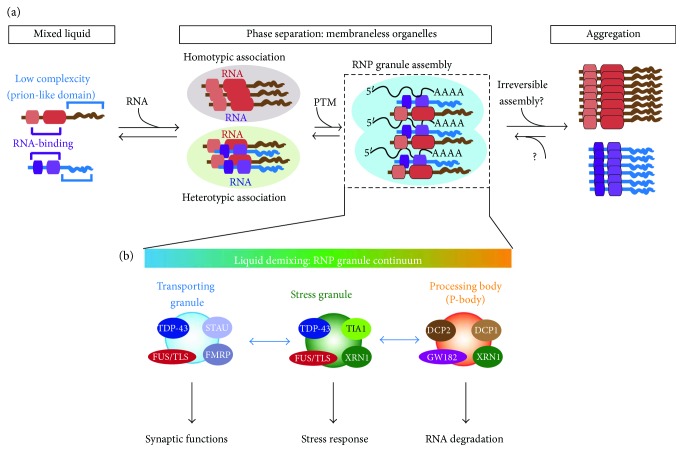
Assembly principle for RNP granules. (a) RNA-binding proteins typically can be separated into RNA-binding domains and low-complexity prion-like domains. Low-complexity domains are intrinsically disordered and can form multivalent weak interactions which nucleate to form phase separation. Posttranslational modifications (PTMs) could aid the occurrence of phase transition. Phase separation generates a phase boundary, such as RNP granules. RNP granules mature into a hydrogel-like state where the molecules have reduced movement. The enrichment of aggregation-prone proteins within the RNP granules may result in the formation of irreversible aggregations observed in neurodegenerative diseases. (b) In neurons, there are three main types of RNA granules: transporting granules, stress granules, and processing body (P-body). Each of them is proposed to perform different functions. Each type of granule can present stereotypical markers but also share common components. Based on the liquid-liquid demixing, the components can easily transition from one type of granule to another as part of the RNP granule continuum.

The discovery that the low-complexity prion-like domains within TDP-43 and FUS self-assemble is rather serendipitous [35, 135]. Unexpectedly, biotinylated isoxazole causes formation of a gel-like precipitate, whose components are RNA-binding proteins resembling those of RNA granules. Inspection of these RNA-binding proteins reveals a common signature: domains of RNA-binding motifs, such as RNA recognition motif (RRM) and KH (hnRNP-K homology) domain, are linked with a low-complexity (LC) domain, which are defined to be sequences in proteins with little amino acid diversity. Such domains are reminiscent of a “prion-like” domain, which are enriched with asparagine, glutamine, tyrosine, and glycine residues [36]. Intriguingly, the structure formed by the LC domains of FUS/TLS is composed of amyloid-like fibers. In contrast to other irreversible pathological amyloid inclusions, amyloid-like fibers formed by RNA-binding proteins are reversible and relatively sensitive to solubilizing reagents, suggesting a fundamental difference between prion-like pathological inclusions and amyloid-like fibers formed by RNA-binding proteins [35]. Using purified recombinant protein fragments with either the prion-like/LC domain or RNA-binding domain, it was demonstrated that the prion-like/LC domain is necessary and sufficient for polymerization. Furthermore, these LC domains from different RNA-binding proteins can coassemble with each other to form either homotypic or heterotypic oligomers, although the affinities between different LC domains may differ [35]. Furthermore, RNA can be recruited via the RNA-binding domain [135]. Taken together, this functional configuration of combining RNA-binding domains and LC domains provides the basis for RNA granule assembly (Figure 3).

In other words, RNA granules are essentially hydrogel-like liquid droplets enriched with RNA and RNA-associated proteins, which may explain how RNA granules could readily switch from between different identities (Figure 3). In fact, RNA granules behaving like liquid droplets have been observed using *in vivo* time-lapse imaging in the germline P granules in *Caenorhabditis elegans* [136]. In somatic cells, including neurons, nontranslating mRNAs, possibly also miRNAs [137], are sorted into different cytoplasmic RNA granules, which can be divided functionally into three different groups: processing bodies (P-bodies), which contain RNA decay machinery [138]; stress granules, which contain translation machinery [139]; and transporting RNP granules, which contain RNAs to be locally translated [140]. Many of the same RNA-binding proteins can be present in different RNA granules [138], suggesting that RNA granules may be dynamically remodeled as part of the RNP granule continuum (Figure 3(b)). Indeed, recent experimental data have shown that the intrinsically disordered domain, that is, the low-complexity prion-like domain, within the RNA-binding proteins can phase separately to form RNA granules *in vitro* and *in vivo* [35, 141–148]. Critically, many of these RNA-binding proteins, including FUS, form more stable amyloid-like structures over time within these liquid droplets [142–145, 147]. Furthermore, disease-causing mutations in TDP-43 and FUS appear to accelerate the aggregation-prone process [142, 145, 147], suggesting a potential link between the reversible hydrogel state and the irreversible pathological fibrillization state. On the other hand, recent work showed that stress-induced phase separation by RNA-binding proteins may be an evolutionally conserved mechanism for cells to adapt and survive environmental stress [149]. Thus, it is tempting to speculate that disease processes may involve hijacking the normal stress adaption response of phase separation into a state favoring the formation of pathological structures formed by the intrinsically disordered domain-containing RNA-binding proteins, such as TDP-43 and FUS.

The concept that membraneless organelles, such as RNA granules, are formed by intrinsically disordered proteins, such as LC-domain-containing RNA-binding proteins, via multivalent weak interactions has now been recognized as an underlying principle for how intracellular compartments can be generated [150, 151]. The phenomenon known as liquid-liquid demixing or phase separation is widely used in assembly of various nuclear bodies, such as nucleoli, gem bodies, Cajal bodies, splicing speckles, and paraspeckles [152], as well as in assembly of intracellular signaling hubs [153]. Intriguingly, two major components of the PSD, SynGAP and PSD-95, have been shown to undergo phase separation. The phase separation process correlates with synaptic activity and is proposed to be a potential mechanism for PSD formation [154]. It is tempting to speculate that this phase separation may underlie the dynamic change of postsynaptic components during synaptic modification. Similarly, a potential phase separation mechanism has been proposed for the clustering of synaptic vesicles in the presynaptic site [126]. Both TDP-43 and FUS are present in axonal and dendritic compartments. FUS has been shown to be part of the PSD, and the level of FUS within the PSD increases in the PSD fraction after neuronal stimulation in culture [115], raising an exciting and open possibility regarding whether the phase separation between PSD modification and RNA granules occurs and whether it could be another mechanism for modifying synaptic strength.

## 5. Conclusions

On the molecular level, TDP-43 and FUS regulate diverse functions affecting RNA metabolism, in particular, splicing and polyadenylation site selection, which take place in the nucleus. The enrichment of TDP-43 and FUS binding to 3′-UTR of cytosolic mRNAs further suggests that TDP-43 and FUS could influence the fates of RNA beyond their nuclear actions. Together, these molecular regulations influence the half-lives, localization, and translation of their target mRNA (Figure 3). As many RNAs involved in synaptic functions are targets of TDP-43 and FUS, it is clear that TDP-43 and FUS could influence synaptic development, maintenance, and plasticity. How to tie in the molecular functions with the pathological findings and symptoms caused by TDP-43 and FUS dysfunctions remains a huge gap. Understanding how TDP-43 and FUS contribute to synaptic function and how synaptic dysfunction is caused by misregulation of these two proteins should be the main focus of the basic research as well as the translational side of ALS-FTD research. With new technologies such as high-throughput and low-input single-cell sequencing and precise genetic manipulation by CRISPR-Cas9, more exciting findings are expected to come.
